# The Obsolescence of Azithromycin for Syphilis Treatment and the Value of Contrasting Different Types of Evidence

**DOI:** 10.1093/ofid/ofae325

**Published:** 2024-06-24

**Authors:** Gustavo Yano Callado, Jorge L Salinas, Alexandre R Marra

**Affiliations:** Faculdade Israelita de Ciências da Saúde Albert Einstein, Hospital Israelita Albert Einstein, São Paulo, Brazil; Division of Infectious Diseases and Geographic Medicine, Stanford University, Stanford, California, USA; Faculdade Israelita de Ciências da Saúde Albert Einstein, Hospital Israelita Albert Einstein, São Paulo, Brazil; Department of Internal Medicine, Carver College of Medicine, University of Iowa, Iowa City, Iowa, USA

We appreciate the comments by Cannon et al regarding our article that compared nonpenicillin antibiotics with benzathine penicillin for syphilis treatment. We conducted a systematic literature review and meta-analysis following rigorous scientific and methodological practices, adhering to standardized guidelines as described in our article [1]. We aimed to summarize the current knowledge through the highest level of medical evidence, such as randomized controlled trials. Although the meta-analysis suggests a potential role for nonpenicillin regimens, we emphasized that we fully agree with the guidelines of the Centers for Disease Control and Prevention recommending penicillin as the first-line option for treating syphilis at any stage [2]. The search for alternative treatments for syphilis seeks to address situations where penicillin cannot be used, such as when there is a shortage of the antibiotic or when the patient is allergic.

Our study presented evidence from randomized clinical trials indicating that azithromycin, doxycycline, and ceftriaxone may be equivalent to penicillin in achieving serologic cure rates after treatment. However, we appreciate Cannon and colleagues for expanding the discussion on why azithromycin should no longer be used as part of programmatic management guidelines despite the results of several published clinical trials. They rightfully point out that since the publication of the trials favoring azithromycin, epidemiologic data have shown a growing spread of azithromycin resistance worldwide. We acknowledge that we could have been more definitive in our discussion and conclusions, stating that although earlier data supported azithromycin, current epidemiologic data now exclude it from treatment guidelines.

Public health and programmatic recommendations should use efficacy data from meta-analyses but also include real-world effectiveness data and surveillance data for rates of drug resistance. We appreciate this opportunity to highlight that although randomized trials and meta-analyses are considered among the highest levels of medical evidence, other kinds of evidence, often regarded as more modest, can be just as informative for treatment recommendations.

## Notes

***Financial support***. None.
